# A Method of Mining Truck Loading Volume Detection Based on Deep Learning and Image Recognition

**DOI:** 10.3390/s21020635

**Published:** 2021-01-18

**Authors:** Xiaoyu Sun, Xuerao Li, Dong Xiao, Yu Chen, Baohua Wang

**Affiliations:** 1 School of Resources and Civil Engineering, Northeastern University, Shenyang 110004, China; sunxiaoyu@mail.neu.edu.cn (X.S.); xuerao_li@163.com (X.L.); 18240439961@163.com (Y.C.); wangbaohua_aiut@163.com (B.W.); 2 Information Science and Engineering School, Northeastern University, Shenyang 110004, China

**Keywords:** open pit mine, loading volume, image recognition, VGG16, least square algorithm

## Abstract

Detection of the loading volume of mining trucks is an important task in open pit mining. Aiming at the addressing the current problems of low accuracy and high cost of the detection of the loading volume of mining trucks, this paper proposes a mining truck loading volume detection model based on deep learning and image recognition. The training and test data of the model consists of 6000 sets of images taken in a laboratory environment. After image preprocessing, the VGG16 network model is used to pre classify the ore images. The classification results are displayed and the possibility of each category is determined. Then, the loading volume of mining trucks is calculated by using the classification results and the least squares algorithm. By using the labeled image data of five kinds of mining truck loading volume, the arbitrary loading volume detection of mining trucks is realized, which effectively solves the problem of a lack of labeled data types caused by the difficulty in obtaining mine data. Root mean square error (RMSE) and mean absolute error (MAE) are used to evaluate the fitting effect of the model. The experimental results show that the model has high prediction accuracy. The average absolute error is 17.85 ${\mathrm{cm}}^{3}$. In addition, this paper uses 400 real mining truck images of open-pit mines to verify the model and the average absolute error is 2.53 $m^{3}$. The experimental results show that the model has good generality and can be applied well to the actual production of open-pit mines.

## 1. Introduction

Loading capacity measurement is a daily production management task in mines [1]. Since there is no online measuring device for the truck loading equipment currently used in the open-pit mines, the output of each part of ore and rock is roughly measured by multiplying the number of carrying vehicles by an agreed single truck loading capacity. This not only seriously distorts the production data of open pit mines, affecting the accurate distribution of ore, but also causes a waste of labor, vehicles and oil [2]. Transportation of ore and rock will have a direct impact on the completion of mine production tasks and the performance appraisal of truck drivers. It is also of great significance to accurately grasp and control the output of each part of ore and rock for the realization of efficient utilization of mineral resources and effective production management.

In addition to the traditional detection methods of manual counting and weighbridge weighing, there are sensor detection methods [3] and material level meter detection methods [4]. Sensor equipment has a high cost and a high failure rate. At the same time, it is greatly affected by severe weather such as rain and snow, dust and fine slag in the mine, which makes daily maintenance inconvenient. The level meter detection method requires the use of multiple level meters and control instruments, which requires a fast response time. At the same time, it is difficult to measure the volume of ore and rock in sheltered transportation vehicles. Therefore, the development of a low-cost, fast and accurate method to detect the loading capacity of mining trucks for the rational mining and utilization of ore and rock is of great significance.

Nowadays, visual sensing technology, image processing, artificial intelligence and other technologies have become more and more popular, the application of computer vision technology and neural network technology in mine production processes has become a new trend. Its non-contact sensing, multi-level information fusion, high-speed modeling calculation and other characteristics meet the requirements of large-scale, uninterrupted and timely feedback of mine production [5]. Tessier et al. [6] put forward a machine vision method to estimate the composition of rock mixtures. Dong et al. [7] used machine vision to realize the ore image segmentation in an ore particle size distribution system. Amankwah et al. [8] used the mean shift and watershed algorithm to segment the ore image and realized the estimation of the size of the ore on the conveyor belt. Patel et al. [9] developed an iron ore online grade monitoring system based on the support vector machine regression (SVR) model. Singh et al. [10] put forward a way of iron-manganese smelter feed ore classification based on the visual texture of manganese-rich, iron-rich, Al_2_O_3_-rich ore and radial basis neural network. Duan et al. [11] improved the U-net network to realize particle detection and contour recognition from images. Hu et al. [12] used a neural network classification algorithm to achieve early detection of mining vehicle faults.

It can be seen that image recognition technology has been widely used in the mining field and has achieved good research results, but research on the detection of the loading capacity of mining trucks is relatively limited. This paper uses numerous images as the research object, and through reasonable image data preprocessing strategy and then through the VGG16 deep neural network model and the least squares method mathematical model [13,14,15] achieves the detection of the loading volume of mining truck.

## 2. Research on Detection Method of Loading Volume of Mine Truck

### 2.1. Traditional Method

In addition to the traditional detection methods of manual counting and weighbridge weighing, there are sensor detection methods and material level meter detection methods. The principle of the sensor detection method is to use a sensor to convert the weight into an electrical signal or a frequency signal, and then process it by a computer to form a control signal or display graphic. The principle of the level meter detection method is that the level meter sends out ultrasonic signal of a certain frequency. When the signal meets the surface of the opposite object, part of the signal is returned back. The distance is calculated according to the propagation speed and propagation time of the ultrasonic wave in the air, which is converted into an electrical signal and sent to the control instrument and then sent to a computer after processing and calculation. These two detection methods have the problems of high equipment cost, high failure rates and inconvenient daily maintenance. Therefore, this paper proposes a data-driven method to detect the loading volume of mining trucks.

### 2.2. Data-Driven Approach

Large-scale labeled data sets are one of the key factors for the great success of deep learning. However, deep learning also has challenges and limitations. Deep learning requires a lot of data and its architecture is also very complex. Even in the same category, thousands of labeled images are needed for deep learning models [16]. In mines, such a large amount of data is difficult to collect and label. Therefore, this paper uses simulated mine car images in a laboratory environment and some real mine car images to verify the model. In addition, this paper combines the VGG16 network in the deep learning algorithm with the least squares method in the data-driven method, using five kinds of labeled image data of mine truck loading capacity (0%, 25%, 50%, 75%, 100%) to realize the detection of arbitrary loading capacity of mining truck. It effectively solves the problem of the lack of labeled data types caused by the difficulty in obtaining mine data.

#### 2.2.1. Deep Learning

In the early deep learning, each layer of the neural network is connected by the way of full connection, which makes the traditional neural network need to train a large number of parameters. At this time, the number of network layers should not be too many, otherwise it will be difficult to train. A convolutional neural network (CNN) is a multi-level and partially connected neural network that simulates the structure of the human brain. It can recognize visual patterns directly from original images through supervised deep learning [17]. A CNN model is generally composed of four layers: convolution layer, pooling layer, full connection layer and softmax classification layer [18]. The connection mode between layers is a non-fully connected convolution calculation. Convolutional neural networks have the ability to extract image features and a deeper CNN usually can extract more specific and complex features. In large data sets such as ImageNet, VGGNet has strong recognition ability, and the effectiveness of feature extraction is proved to be effective on large data sets [19].

VGGNet is a deep neural network model proposed by Simonyan and Zisserman of the Oxford University [19]. The model achieves higher precision and higher efficiency prediction tasks by increasing the number of network layers. Zhou et al. [20] constructed a classification network of 10 tomato organs on VGG16, and the average detection accuracy of fruits, flowers and stems were 81.64%, 84.48% and 53.94%, respectively. Guo et al. [21] used VGGNet to build a network suitable for skin damage classification. Ke et al. [22] proposed a lightweight VGGNet epilepsy EEG recognition method based on global MIC. These studies show that VGGNet has good application prospects in image classification.

#### 2.2.2. Least Squares Regression

When the mechanism of a process object is not clear, the method of data-driven modeling can be used to build the model. Data-driven modeling refers to mining useful information for modeling from the input and output data of the controlled system, and using this information to establish the mathematical relationship between the dominant variable and the auxiliary variable. According to whether the process object has nonlinearity, the data-driven modeling method can be divided into linear regression method, artificial neural network method, support vector machine method and fuzzy modeling method [23].

The least squares method [24,25] is one of the linear regression methods. It finds the best function matching of data by minimizing the sum of squares of errors. Subsequently, through the research of many scholars, the least square method is widely used in various fields, and a series of extended algorithms are proposed. Fisher et al. [26] studied the multivariate total least squares algorithm (MTLS) based on the SVD method, which turned the observation vector and the parameter vector to be estimated into a matrix form. Rao [27] studied the scaled total least squares algorithm (scaled TLS = STLS) under the condition that the error in the pre-test unit weights of the observation value and the coefficient matrix is not equal or the observation value error is not equal to the coefficient matrix error. Schaffrin et al. [28] studied the overall least square estimation algorithm with equality constraints (equality-constrained TLS, ECTLS). Zhang et al. [29] further studied the inequality-constrained TLS (ICTLS) algorithm with inequality constraints. Yang et al. [30] combined the total least squares fitting method with the feature point extraction method to obtain and vectorize the target residential block. Chen et al. [31] applied the total least squares method to the calculation of space resection, and obtained more reasonable and reliable exterior azimuth elements. Yuan et al. [32] used the TLS method to analyze and compare the applicability of the similarity transformation model in the map scanning digitization process.

## 3. Mining Truck Loading Volume Detection Method Based on VGG16 and Least Square Algorithm

### 3.1. Image Preprocessing

Image preprocessing is significant for image recognition. The main purpose of image preprocessing is to reduce the irrelevant information in the image, improve the efficiency of feature extraction, and at the same time can greatly simplify the data, improve the detection accuracy and detection speed. Moreover, due to the large dust in the mine and the complicated background, which increases the difficulty of image feature extraction. Therefore, it is very important for image preprocessing. This paper adopts two image preprocessing strategies: pixel value reduction and histogram equalization. Excessive pixel values will increase the processing time in network training, while reducing the pixel value can greatly improve the overall detection efficiency of the model. In the whole data set, histogram equalization is used to enhance the foreground background contrast, which can increase the contrast between ore and tramcar without losing the overall contrast. At the same time, some details of the image can be highlighted.

### 3.2. Detection Model of Loading Volume of Mine Truck

#### 3.2.1. VGG16 Model

**Figure 1:**
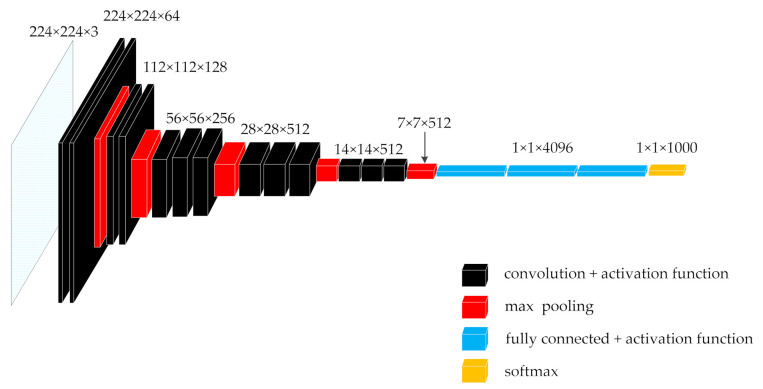
VGG16 network structure diagram.

The input of the VGG16 network is a fixed-size RGB 2D image, followed by a series of stacked convolutional layers with a core size of 3 × 3. Each two or three consecutive stacked convolution layers is a small unit module of a network, named block. A Max-pooling layer is connected to each block to reduce the size of the input image and maintain the translation invariance of the network. After multiple stacked block cells, the output will be connected to a three-layer traditional neural network, that is, the three-layer full connection layer. The final classification output is a softmax multi-classifier. The VGG16 network structure is shown in Figure 1 [19].

In the continuously stacked local block unit model, the core size of each convolution layer is 3 × 3, and the sliding step size of the core is 1. Before convolution operation of input, the input peripheral must be padded with the size of 1. There is no Max-pooling layer between consecutive convolutional layers in the local block unit, but a Max-pooling layer is connected after each block unit.

**Figure 2:**
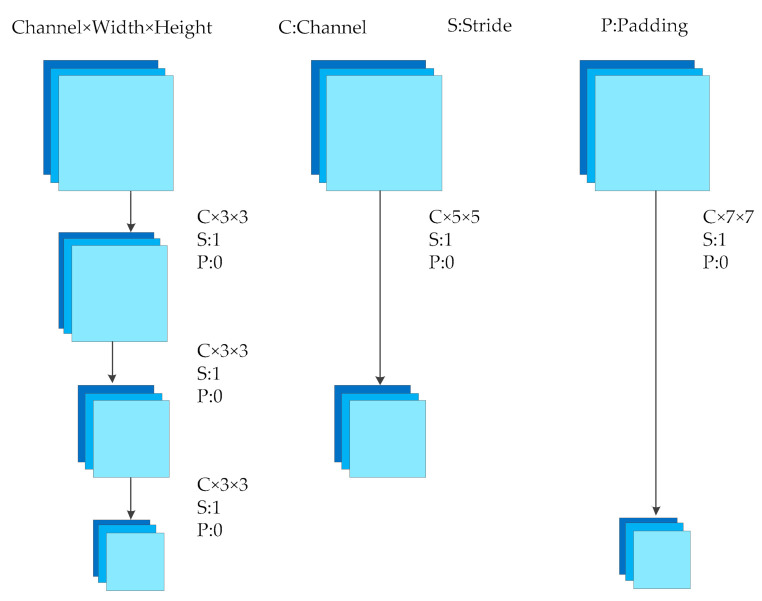
Different size convolutions and stacked convolutions.

Continuously stacked convolutional layers all use a 3 × 3 size convolution core, which is the smallest convolution core that can cover the input upper left, upper right, lower left, lower right, and upper, lower, middle, left and right directions. In this way, the input can be convoluted more intensively and more features can be extracted. Moreover, by stacking multiple convolution layers of 3 × 3 convolution cores, it can be equivalent to a convolution layer with 5 × 5 convolution cores or 7 × 7 convolution cores, as shown in Figure 2. However, the stacking of multiple small-core convolutional layers will have a better nonlinear conversion effect than a single convolutional layer of a large-dimensional convolution core, and it has better ability to extract key features.

The activation function is connected to the back of each convolutional layer and the fully connected layer to perform non-linear mapping calculations. Dropout technology is used behind the first and second full connection layers to improve the network generalization ability and prevent the network from over fitting.

The specific network configuration is shown in Table 1 [19].

#### 3.2.2. Least Squares Mathematical Model

The least squares algorithm [33] is a basic parameter estimation method based on the least squares sum of errors. Its basic idea is to minimize the sum of squares of the difference between the model output and the measured output with the estimator [34]. The least squares method is one of the most widely used curve fitting methods. It has very important significance in theoretical research and engineering applications. At the same time, it is the basis of many other more complex methods.

The least squares method in the MATLAB simulation environment provides a variety of methods for fitting. We can not only use function methods to fit, such as the polyfit function and lsqcurvefit function, but also use graphical interface fitting and the cftool curve fitting toolbox.

#### 3.2.3. Mining Truck Loading Volume Detection Model Based on VGG16 and the Least Squares Algorithm

**Figure 3:**
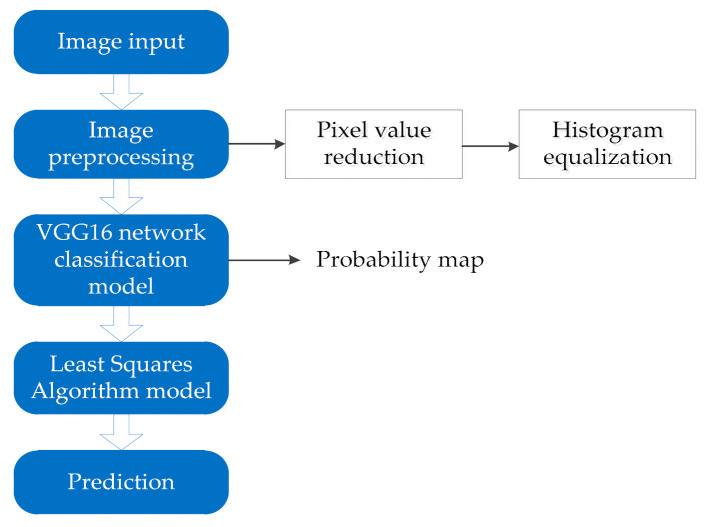
Algorithm flowchart.

The detection model consists of two parts: one part is an image classification model based on the VGG16 deep neural network and the other part is a mine truck loading volume prediction model based on the least squares algorithm. The image is classified by the VGG16 deep neural network and the output result is the probability value of each predicted category. The classification result of the image classification prediction and the corresponding probability value are used as the data to be fitted by the least squares method, and the output result is the predicted value of the loading volume of the mining truck. The loading volume detection process of mining truck is shown in Figure 3.

The VGG16 deep neural network is applied to preliminarily classify the images and realize the classification of the loading volume of mining trucks. Five kinds of images are taken as the input of the network and the corresponding ore loading label is formulated as the network output. The VGG16 deep neural network contains multiple convolution segments, which are suitable for processing image data. The original image is extracted through the convolution kernel and the characteristics of the pooling layer are summarized, and then forwarded layer by layer. At the same time, the output error is passed back layer by layer to update the weight threshold of the network, and finally the optimization of the network structure is realized, that is, the output error is minimized.

The image is classified by VGG16 deep neural network, and the output result is the probability value of each predicted category. The classification result of the image classification prediction and the corresponding probability value are used as the data to be fitted by the least squares method and the output result is the predicted value of the loading volume of the mining truck. The least squares model selects the first two classification results with the highest probability among the classification results for curve fitting. p00, p10 and p01 in the following formula are the three coefficients to be fitted.

The fitting formula is expressed as:

(1) $$V=\left(p00+p10x+p01y\right)\times V_{m}\;x=C_{1}P_{1}\;y=C_{2}P_{2}$$

Among them, *V* indicates the predicted loading volume; *V_m_* indicates the maximum loading volume of minecart; *C*_1_ indicates the maximum probability category; *C*_2_ indicates the second most probable category; *P*_1_ indicates the probability of the highest probability category; *P*_2_ indicates the probability of the second highest probability category.

### 3.3. Evaluation Method

The neural network structure parameters affect the prediction performance of the network. However, there is no specific theory to help neural networks determine effective parameters for different data samples. Among them, the learning rate is one of the most important parameters, which determines whether the objective function can converge to a local minimum and when to converge to the minimum. A proper learning rate can make the objective function converge to a local minimum in a short time. An incorrect learning rate may cause the network to converge too slowly or oscillatingly, or even fail to converge. Meanwhile, with the massive growth of data sets and memory limitation, it is more and more infeasible to load all the data into the network at one time, so the number of samples sent into the network each time directly affects the accuracy of model classification and the overall training time. Therefore, the choice of BatchSize is also crucial. In addition, the use of non-linear activation functions makes deep neural networks have stronger expressive capabilities. Choosing the appropriate activation function can improve the learning speed of the network. Therefore, this paper uses the control variable method to conduct an enormous number of network topology generation experiments from the learning rate, BatchSize, and activation function to determine the network structure parameters in order to achieve better network performance. In this process, the multiple correlation coefficient (R) and root mean square error (RMSE) are selected as the indexes to evaluate the performance of the model. *R* is an index to evaluate the quality of fitting. The correlation between fitting results and measured values is reflected by the correlation coefficient between fitting results and measured values. The closer the value of *R* is to 1, the better the fitted regression equation. *RMSE* reflects the size of the error between predicted data and actual data. The closer the *RMSE* is to 0, the better the fitted regression equation. The calculation formula of *R* is given by Equation (2), and the calculation formula of *RMSE* uses Equation (3).

(2) $$R=1-\frac{\sum_{i=1}^{L}{\left({\hat{t}}_{i}-t_{i}\right)}^{2}}{\sum_{i=1}^{L}{\left(t_{i}-\bar{t}\right)}^{2}}$$

Among them, $t_{i}$ indicates the original regression value, $\bar{t}$ indicates the average of the original regression value, ${\hat{t}}_{i}$ indicates the predicted regression value, L indicates the number of samples:

(3) $$RMSE=\sqrt{\frac{1}{L}\overset{L}{\underset{i=1}{\sum }}{\left({\hat{t}}_{i}-t_{i}\right)}^{2}}$$

Among them, $t_{i}$ indicates the original regression value, ${\hat{t}}_{i}$ indicates the predicted regression value, L indicates the number of samples.

## 4. Experimental Process

### 4.1. Deep Learning Framework and Hardware Platform Environment

Tensorflow is the second generation of artificial intelligence learning system developed by Google based on Dist Belief. It is widely used in speech recognition, natural language processing, computer vision and so on. It is an open source software library for numerical computation using data flow graphs. It was originally developed by researchers and engineers of Google Brain group for machine learning and neural network research. The experiment in this article is based on the Tensorflow deep learning framework, and is performed on a platform configured with Windows 10, an Intel^®^ Core™ i5-8400 CPU @ 2.80 GHz processor and a memory of 16.00 GB.

### 4.2. Image Acquisition and Preprocessing

**Figure 4:**
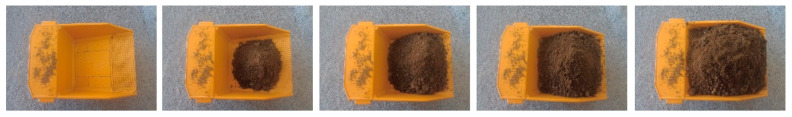
Part of the training set images.

**Figure 5:**
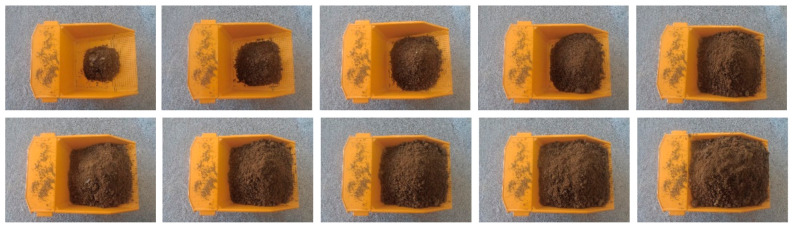
Part of the test set images.

**Figure 6:**
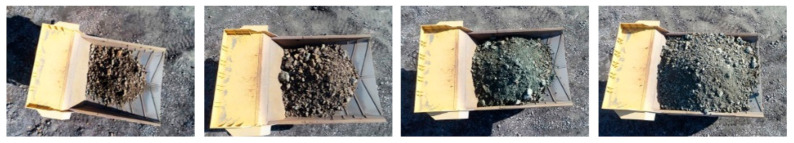
Part of the supplementary test set images.

In practical application, the camera is installed in the mine crushing station to detect the loading volume of the mining truck, and the shooting angle is from the top. However, due to the difficulty in obtaining mine images and corresponding tag values of arbitrary loading amount of ore and rock for training and testing. Therefore, this paper uses simulated mine car images in a laboratory environment to verify the model and the viewing angle is a fixed height 22 cm overhead view. According to on-the-spot observation in the open-pit mine and some image collection and collation, it is found that in the actual production operation of the open-pit mine, electric shovels are used to load ore and rock into the mining trucks. Therefore, five types of images can be collected, which are empty truck (loading volume 0%), loading one shovel (loading volume 25%), loading two shovels (loading volume 50%), loading three shovels (loading volume 75%) and loading four shovels (loading volume 100%). Therefore, this paper uses five kinds of labeled image data of mine truck loading capacity (0%, 25%, 50%, 75%, 100%) to realize the detection of arbitrary loading capacity of mining truck, and effectively solves the problem of less labeled data types caused by the difficulty in obtaining mine data. In order to ensure the authenticity of the experiment, the data set used in this paper is composed of 6000 images. Among them, the training set is composed of five categories of images: empty vehicle (loading volume 0%), loading volume 25%, loading volume 50%, loading volume 75%, full vehicle (loading volume 100%), 1000 images of each category. The test set is composed of 10 kinds of images: loading volume 10%, loading volume 20%, loading volume 30%, loading volume 40%, loading volume 50%, loading volume 60%, loading volume 70%, loading volume 80%, loading volume 90%, loading volume 100%, 100 images of each category. The data sets in laboratory environment were made by measuring cup, sand and mining truck model. The size of the mining truck model is 190 mm × 112 mm × 100mm, the bucket capacity is 300 ${\mathrm{cm}}^{3}$, and the shooting height is 22 cm. The mining truck photographed in the actual open-pit mine is a Komatsu HD785 mining truck, the size is 10,600 mm × 5700 mm × 5300 mm, the bucket capacity is 60$\;m^{3}$, and the shooting height is 11 m. The indicators mentioned above are all in an about 50 times ratio relationship, which can participate in the verification of the model. Some images of training set and test set are shown in Figure 4 and Figure 5. In order to verify the universality of the model, four kinds of real mine car images are collected as supplementary test sets to verify the model. The supplementary test set is composed of four categories of images: loading volume 25%, loading volume 50%, loading volume 75%, loading volume 100%, 100 images of each category. Some images of the supplementary test set are shown in Figure 6.

**Figure 7:**
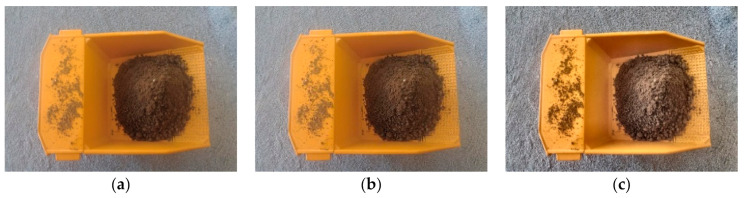
Images after preprocessing: (**a**) Original image; (**b**) Pixel value reduction; (**c**) Histogram equalization.

This paper adopts two image preprocessing strategies: pixel value reduction and histogram equalization. The original image is in RGB format with a pixel size of 1280 × 960. Reducing the pixel value can greatly improve the overall detection efficiency of the model. Therefore, the pixel value reduction is used to convert the original image into an image with a pixel value of 224 × 224, as shown in Figure 7b. In the whole data set, histogram equalization is used to enhance the foreground background contrast, which can increase the contrast between ore and tramcar without losing the overall contrast. At the same time, some details of the image can be highlighted, as shown in Figure 7c.

### 4.3. Production of TFRecords Data Sets

(1) Creation of TFRecords data

The TFRecords file contains tf.train.Example Protocol buffer, data can be filled into the Example protocol buffer. The protocol buffer is then serialized into a string and passed through the tf.python_ io.TFRecordWriter class to the TFRecords file.

(2) Reading TFRecords data

First, we create a data flow diagram. This data flow diagram consists of a number of pipeline stages, which are connected by queues. The first stage will generate file names, read these file names and put them in the file name queue. The second stage reads data from the file, generates samples, and places the samples in a sample queue. Depending on the settings, you can also copy the samples of the second stage to make them independent of each other, so that they can be read from multiple files in parallel. At the end of the second stage, queue operation is performed, that is, join the queue and leave the queue in the next stage. Because we want to start running these queued operations, the training loop will dequeue samples in the sample queue continuously.

### 4.4. The Establishment of Model

The detection model is mainly composed of two parts: the first part is an image classification model based on the VGG16 deep neural network, and the second part is a mine truck loading volume prediction model based on the least square algorithm.

#### 4.4.1. VGG16 Deep Neural Network Model

The choice of hyperparameters of activation function is of great significance to neural networks. Therefore, this paper uses the control variable method to conduct an enormous number of network topology generation experiments from the learning rate, BatchSize, and activation function to determine the network structure parameters in order to achieve better network performance. In this process, the multiple correlation coefficient (R) and root mean square error (RMSE) are selected as the indexes to evaluate the performance of the model. 

The learning rate has an impact on the speed at which the model converges to a local minimum. Therefore, choosing an appropriate learning rate means that the model can be trained in a shorter time. An artificial neural network with an incorrect learning rate may require a very high number of iterations to converge, or even cause the network to fail to converge. Table 2, Table 3 and Table 4 record the effect of VGG16 network on image data training in laboratory environment under different learning rate, BatchSize, and activation function, from which the optimal network parameters can be obtained. The bucket capacity of the mining truck model is 300 ${\mathrm{cm}}^{3}$. Table 2 shows the performance of VGG16 networks with different learning rates. The model with a learning rate of 0.003 has the lowest *RMSE* of 2.70 ${\mathrm{cm}}^{3}$ and an *R* value of 0.9990.

The non-linear activation function makes the neural network more expressive and can better fit the objective function. For deep neural network networks, commonly used activation functions are Sigmoid, Relu, Elu, and so on. This paper conducts experiments on 6 different activation functions. It can be seen from Table 3 that the *RMSE* and *R* of networks with different activation functions are very different. It is worth noting that the Elu function can greatly improve the prediction performance.

Batchsize is an important parameter in machine learning. Selecting the appropriate batchsize can effectively improve the memory utilization and more accurately move towards the direction of extreme value. An incorrect BatchSize value may cause the network to fall into a local optimum or fail to converge. As shown in Table 4, choose different BatchSize to develop and train the VGG16 network separately. When the BatchSize is 20, the maximum *R* and minimum *RMSE* are available.

In summary, through multiple learning training and result analysis, the optimal network structure parameters are selected: learning rate 0.003, Elu activation function, and BatchSize of 20. At this time, the network fitting degree is high, and the prediction performance of the model is good.

#### 4.4.2. Least Squares Mathematical Model

**Figure 8:**
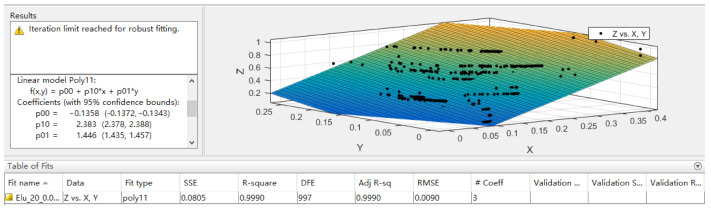
Least squares algorithm fitting effect diagram.

The image is classified by VGG16 deep neural network and the output result is the probability value of each predicted category. The classification result of the image classification prediction and the corresponding probability value are used as the data to be fitted by the least square method, and the output result is the predicted value of the loading volume of the mining truck. The least squares model selects the first two classification results with the highest probability among the classification results for curve fitting. In this paper, the cftool fitting toolbox in MATLAB is used for curve fitting of least square method. The fitting effect diagram is shown in Figure 8.

Therefore, the best fitting parameters in Equation (1) are as follows:

p00=−0.1358p10=2.383p01=1.446

## 5. Experimental Results and Discussions

The mean absolute error (MAE) and root mean square error (RMSE) were calculated to evaluate the performance of the model. The mathematical formula of RMSE is shown in Equation (3) and the mathematical formula of MAE is as follows:

(4) $$MAE=\frac{1}{L}\overset{L}{\underset{i=1}{\sum }}|\left({\hat{t}}_{i}-t_{i}\right)|$$

where ${\hat{t}}_{i}$ indicates the predicted value and $t_{i}$ indicates the measured value, L indicates the number of samples.

**Figure 9:**
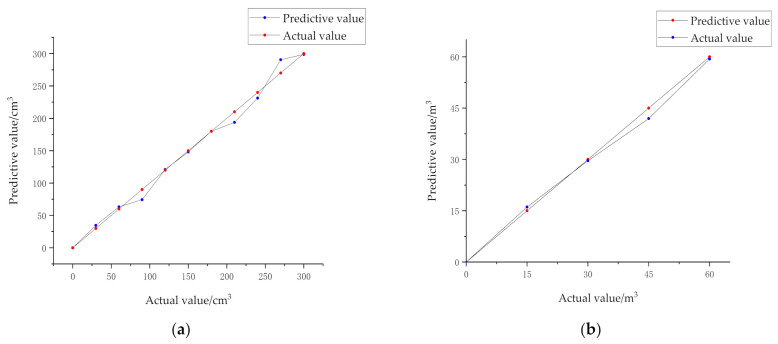
Actual value-predictive value line chart: (**a**) Data in a laboratory environment; (**b**) Data in an open-pit mine.

Figure 9 shows the detection accuracy of the mining truck loading volume detection model established in this paper. The blue line represents the actual loading volume, and the red line represents the predicted loading volume. Figure 9a shows the detection results of 10 categories of mining trucks in a laboratory environment, and Figure 9b shows the detection results of four categories of real mining trucks in an open-pit mine. It can be seen from the figure that the predicted values are distributed near the corresponding actual values and the distance is small. Therefore, the prediction effect of the model is more accurate.

**Figure 10:**
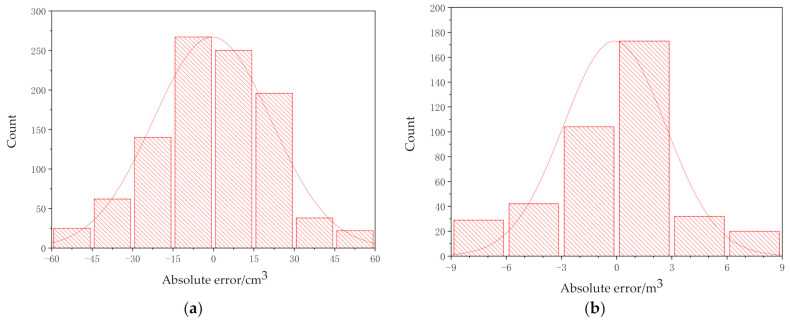
Error distribution histogramt: (**a**) Data in a laboratory environment; (**b**) Data in an open-pit mine.

Figure 10a is the error distribution histogram of the detection results of 10 categories of mining trucks in a laboratory environment, and Figure 10b is the error distribution histogram of the detection results of the four categories of real mining truck images in the open-pit mine. As shown in Figure 10, the histogram of test error distribution of the model established in this paper is close to symmetric normal distribution, and its shape is high in the middle, and gradually decreases on both sides. It can be clearly seen from the figure that the detection error of mining truck model is between −30 ${\mathrm{cm}}^{3}$ and 30 ${\mathrm{cm}}^{3}$, and the detection error of real mining truck is between −3 $m^{3}$ and 3 $m^{3}$, which error distribution histogram indicates that there are fewer outliers and the model has strong stability.

**Figure 11:**
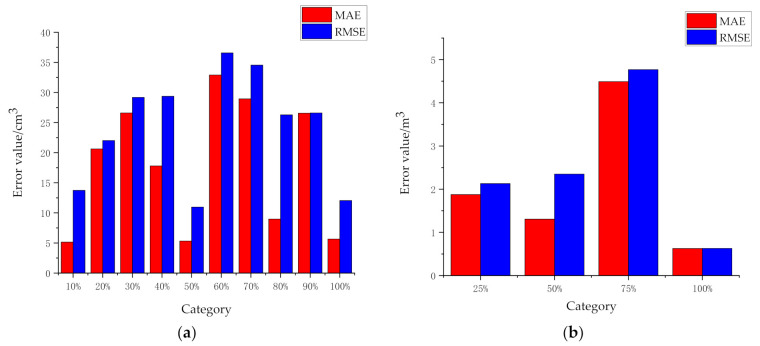
Model performance index histogramt: (**a**) Data in a laboratory environment; (**b**) Data in an open-pit mine.

Figure 11 shows the performance indicators of the model: mean absolute error (MAE) and root mean square error (RMSE). Figure 11a is the evaluation index of 10 categories of mining truck detection results in a laboratory environment, and Figure 11b is the evaluation index of four categories of real mine truck image detection results in open-pit mines. MAE is the average value of absolute error, which can better reflect the actual situation of prediction error. RMSE is the square root of the ratio of the sum of squares of the deviation between the observed value and the true value and the square root of the ratio of the number of observations L, which is used to measure the deviation between the observed value and the true value. From the overall point of view in Figure 11, MAE and RMSE values are small, and the performance of the model established in this paper is good. 

## 6. Conclusions

In order to introduce artificial intelligence technology and image recognition technology into the detection of mining truck loading volume, this paper proposes a detection method of mining truck loading volume based on deep learning and image recognition. Our conclusions are summarized as follows:

(1) A method for detecting the loading volume of mining trucks based on image recognition is proposed. The main steps include using pixel value reduction and histogram equalization two image preprocessing strategies for the image. Then the VGG16 deep neural network model is used to pre-classify the images, and the classification results are displayed and the possibility of each category is determined. Finally, the loading volume of mining truck is calculated by the classification results and the least squares algorithm of a data-driven modeling method.
(2) The model is verified by using a large number of image data taken in the laboratory environment and real mine car images. The average error is 17.85 ${\mathrm{cm}}^{3}$ and 2.53 $m^{3}$, respectively. The error is small, which proves that the proposed method has high prediction accuracy and versatility.
(3) One of the innovative points of this paper is to combine the deep learning model with the mathematical model, using the labeled image data of five kinds of mining truck loading capacity, whereby the arbitrary loading capacity detection of mining truck is realized. It effectively solves the problem of a lack of labeled data types caused by the difficulty of data acquisition in mines.
(4) The second innovation of this paper is that the artificial intelligence technology and image recognition technology are introduced into the detection of mining truck loading volume. This method has the advantages of low cost, greatly reducing the waste of resources and the use of human and material resources, and improving the degree of detection automation.

## Figures and Tables

**Table 1:** Parameter configuration of VGG16 network model.

	Convolution Core 3 × 3	Convolution Core 3 × 3	Convolution Core 3 × 3	Pooling Layer	Output Size
input	224 × 224 × 3				
Block 1	Stride:1 Padding:1 Core size:3 Core num:64	Stride:1 Padding:1 Core size:3 Core num:64	------	Type:Max Stride:2 Core size:2	224 × 224 × 64
Block 2	Stride:1 Padding:1 Core size:3 Core num:128	Stride:1 Padding:1 Core size:3 Core num:128	------	Type:Max Stride:2 Core size:2	112 × 112 × 128
Block 3	Stride:1 Padding:1 Core size:3 Core num:256	Stride:1 Padding:1 Core size:3 Core num:256	Stride:1 Padding:1 Core size:3 Core num:256	Type:Max Stride:2 Core size:2	56 × 56 × 256
Block 4	Stride:1 Padding:1 Core size:3 Core num:512	Stride:1 Padding:1 Core size:3 Core num:512	Stride:1 Padding:1 Core size:3 Core num:512	Type:Max Stride:2 Core size:2	28 × 28 × 512
Block 5	Stride:1 Padding:1 Core size:3 Core num:512	Stride:1 Padding:1 Core size:3 Core num:512	Stride:1 Padding:1 Core size:3 Core num:512	Type:Max Stride:2 Core size:2	14 × 14 × 512 7 × 7 × 512
Fully connected layer-1		Output node num: 4096			1 × 1 × 4096
Fully connected layer-2		Output node num: 4096			1 × 1 × 4096
Fully connected layer-3		Output node num: 1000			1 × 1 × 1000
Softmax					

**Table 2:** The effect of learning rate on the model.

Learning Rate	*R*	*RMSE* $\left(cm^{3}\right)$
0.001	0.9972	4.56
0.003	0.9990	2.70
0.005	0.2429	75.09
0.007	0.0064	86.28
0.01	0.2831	73.08
0.1	−4.885	86.37

**Table 3:** The effect of activation function on the model.

Active Function	*R*	*RMSE*
Tanh	0.7157	45.87
Sigmoid	−3.7470	86.37
Relu	0.4576	63.57
Elu	0.999	2.70
Leaky Relu	−4.885	86.37
Selu	0.7993	37.62

**Table 4:** The effect of Batchsize on the model.

BatchSize	*R*	*RMSE*
10	0.9975	4.32
20	0.9990	2.70
30	0.9984	3.69
40	0.9956	5.70
50	0.9961	5.37
100	0.9980	3.90

## Data Availability

Not applicable.
